# College Graduates

**Published:** 1895-05

**Authors:** 


COLLEGE GRADUATES.—The multitude of physicians turned out by American medical colleges shows great industrial enterprise. Great Britain has one physician to every 1,707 of the population; Germany one to every 3,038; France one to every 2,766; Austria one to every 3,857; Belgium one to every 2,841; Italy one to every 3,536; and Spain one to every 3,375. In the United States there are about 100,000 physicians, or one to every 626 of the population, and still they come. It is indeed inspiring to think of the higher moral and philanthropic tone here prevailing that finds such eloquent utterance in the great annual volunteer army devoted to the cause of man's physical salvation. Surely the mills of the medical gods grind neither slow nor fine, but it will not be denied that they grind with vigor and with unbounded faith in the assimilable powers of the American profession.

